# SMapper: visualizing spatial prevalence data of all types, including sparse and incomplete datasets

**DOI:** 10.1093/bioadv/vbad176

**Published:** 2023-12-01

**Authors:** Lynn Khellaf, Arwin Ralf, Khanh Toan Nguyen, Manfred Kayser, Michael Nothnagel

**Affiliations:** Cologne Center for Genomics, University of Cologne, 50931 Cologne, Germany; Department of Genetic Identification, Erasmus MC University Medical Center Rotterdam, 3015 GD Rotterdam, The Netherlands; University Hospital Cologne, 50937 Cologne, Germany; Department of Genetic Identification, Erasmus MC University Medical Center Rotterdam, 3015 GD Rotterdam, The Netherlands; Cologne Center for Genomics, University of Cologne, 50931 Cologne, Germany; University Hospital Cologne, 50937 Cologne, Germany

## Abstract

**Motivation:**

We introduce SMapper, a novel web and software tool for visualizing spatial prevalence data of all types including those suffering from incomplete geographic coverage and insufficient sample sizes. We demonstrate the benefits of our tool in overcoming interpretational issues with existing tools caused by such data limitations. We exemplify the use of SMapper by applications to human genotype and phenotype data relevant in an epidemiological, anthropological and forensic context.

**Availability and implementation:**

A web implementation is available at https://rhodos.ccg.uni-koeln.de/smapper/. A stand-alone version, released under the GNU General Public License version 3 as published by the Free Software Foundation, is available from https://rhodos.ccg.uni-koeln.de/smapper/software-download.php as a Singularity container (https://docs.sylabs.io/guides/latest/user-guide/index.html) and a native Linux Python installation.

## 1 Introduction

Visualizing spatial prevalence data, where the occurrence of a particular trait or feature may vary between populations, both human and nonhuman, residing in different geographic regions, is relevant in fields as diverse as genetics, evolution, epidemiology, public health, anthropology, ecology, forensics, and others. Several software tools exist for this purpose, such as Heatmapper (Babicki *et al.* 2016), GeoDa (Anselin *et al.* 2006), and Generic Mapping Tools (Wessel *et al.* 2019), as well as general-purpose geographic information system tools, such as QGIS (https://www.qgis.org/), ArcGIS (https://www.arcgis.com/), and StatPlanet (http://www.statsilk.com/software/statplanet). These tools generate simple heatmaps for illustrating spatial distribution of prevalence data, but implicitly assume large-enough underlying datasets of sufficient geographic coverage. However, most real-world prevalence datasets have data points based on small sample sizes and are of incomplete geographic coverage. Spatial map graphs produced from such limited data with available software tools are susceptible to various misinterpretation issues. As common with existing tools, the inability of differentiating between missing data and zero-frequency data can lead to severe misinterpretations of the true spatial distributions. Small-sized sample data, inducing increased variance of prevalence estimates and increased risk of a downward bias to zero in these estimates due to a noncapture of low-prevalence, can cause spurious impressions of a strongly localized spatial distribution with available tools. Furthermore, existing tools often assign prevalence data to political or governmental units, such as countries or districts, whereas the underlying feature prevalence distribution does not strictly follow such units. A prominent example relevant in population genetics, anthropology and forensics is the genetic inference of paternal and maternal biogeographic ancestry (BGA) based on the spatial distribution of Y-chromosomal and mitochondrial DNA haplogroup frequency data. Existing tools that do not account for limited geographic coverage and small sample sizes may spuriously pinpoint to a narrow region of paternal and maternal BGA, whereas in reality, the haplogroup exists in a much wider geographic region at low frequency not revealed by the tool due to data limitations. A resulting grave consequence could be, for instance, a wrong incrimination of a minority group in a police investigation to search for an unknown perpetrator, who may not be found with such approach. To overcome interpretation issues with existing tools caused by sparse and incomplete spatial prevalence data, we introduce the novel web and software tool SMapper.

## 2 Methods

To overcome previous tools’ shortcomings in adequately representing the underlying data limitations, we have developed a novel user-friendly publicly available web implementation and software, SMapper, to visualize spatial prevalence data of all types, including those that are sparse, have limited geographic coverage and were generated from small sample sizes.

*Design principles*. SMapper combines two layers to indicate missing data as well as estimation of uncertainty. The *first layer* distinguishes zero-frequency estimates (ZFE) from missing data by clearly marking the latter. Instead of an easily ignorable, homogeneous background, missing data are indicated by hachures, strongly signaling data absence. ZFEs are interpolated based on the inverse of the sample size as an approximation of the upper limit for the feature prevalence at any given data point, and appear grey-shaded, where darker grey indicates a higher confidence based on larger sample size. The *second layer* presents interpolated nonzero frequency (NZF) along an adjustable yellow-red color gradient, indicating near-zero (bright yellow) up to high (red) prevalence. Under the assumption that the prevalence follows a smooth distribution, values are intentionally presented solely with respect to geography, leaving out political borders of countries or districts, to avoid spurious differences and potential misinterpretations.

*Input and output*. Input conforms to a generic format (Supplementary Table S1) allowing for multiple sampling sites and features with absolute counts. Sites are assigned to polygons by name. SMapper produces graphic files in *png* and *pdf* format, separately for each feature listed in the input file, complemented by layer-specific legends. All output is included in a single zip file for the user’s convenience.

*Algorithm*. Based on public domain polygon vector data (https://www.naturalearthdata.com/; see SI), interpolation smoothing around data points inversely correlates with their sample size for each layer. SMapper allows multi-threading and runs a GUI enabling easy manual and automated correction of polygon names not correctly specified in the input. See Supplementary Information for details. SMapper has been implemented as a web tool as well as a stand-alone software in Python.

## 3 Application examples

**Figure 1.**
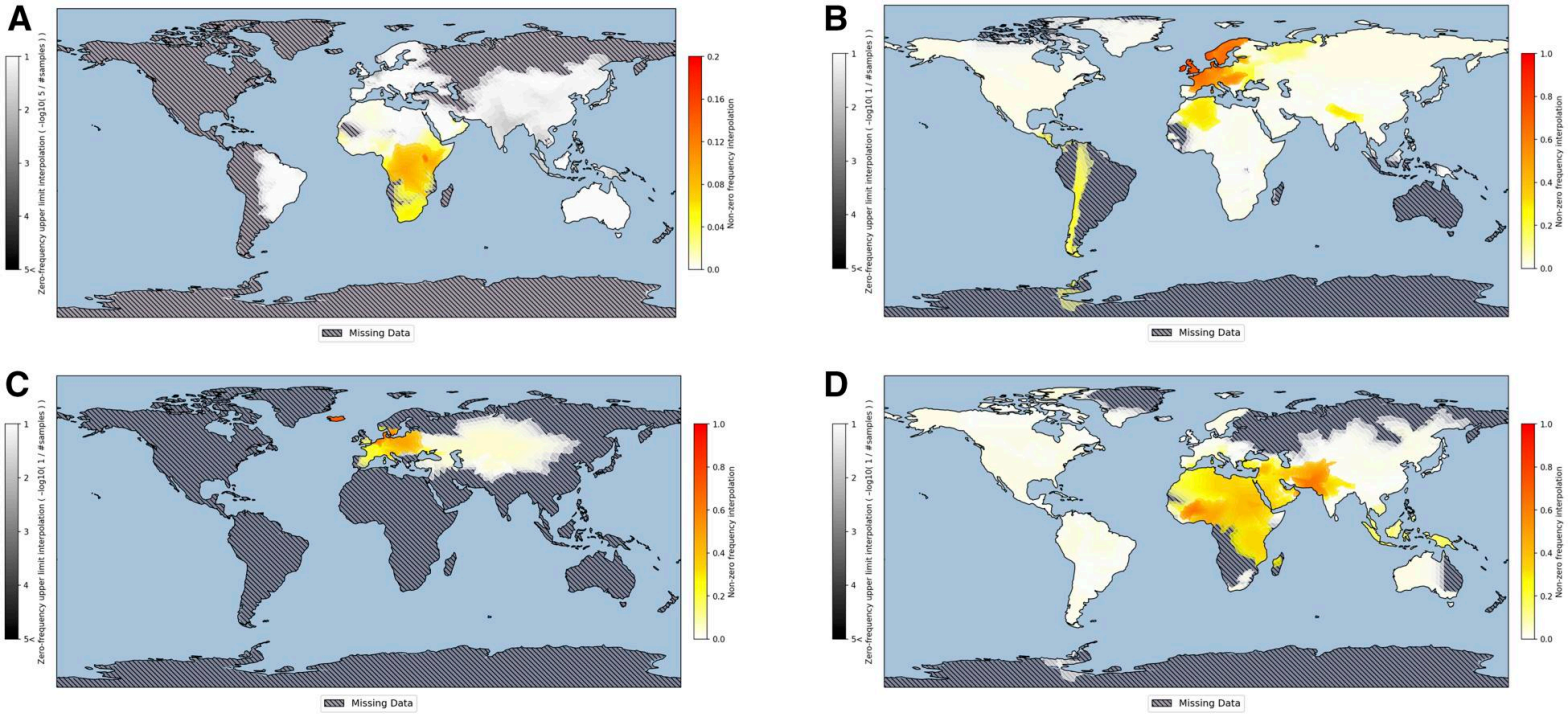
Examples of SMapper-based global spatial prevalence visualizations of human genotype or phenotype data. (A) Map for Y-haplogroup E-M75 (E2); (B) map for the lactase-persistence conferring allele of SNP rs4988235; (C) map for blue eye color; (D) map of consanguinity rates. Interpolated NZF estimates are presented along a yellow-red gradient whereas interpolated ZFE estimates are grey-shaded by the inverse of the sample size. Regions with absent data are clearly marked as hatched. Note the different NZF scales used in the panels.

SMapper allows visualizing any type of spatial prevalence data, limited or not, on a global scale. For illustration purposes, we applied SMapper to four example datasets, including haplogroup Y-haplogroup E-M75, the lactase-persistence conferring rs4988235 allele (Anguita-Ruiz *et al.* 2020), blue eye color (Katsara and Nothnagel 2019), and consanguinity (Bittles and Black 2010) in human populations (Fig. 1). These maps demonstrate the benefits of SMapper over previously developed mapping tools. In particular, they clearly indicate regions with missing information and those covered by only small sample sizes alongside frequency-interpolated areas irrespective of political borders. In this way, SMapper helps to avoid misinterpretations of a spuriously localized occurrence of a data feature by drawing attention to regions that have not been well covered (Fig. 1A), clearly indicates that large or even extremely large areas may have not or not well been covered (Fig. 1B and C), and avoids impressions of possibly spurious country differences as well as potentially spurious regression-model baseline value presentations. Note that in Fig. 1D the ZFE layer is virtually absent since all studies reported nonzero consanguinity rates. See Supplementary Figs 1–4 for a direct comparison of SMapper with Heatmapper for these four examples. Please note that some example datasets intentionally contain some geographic designations that are not contained in the polygon vector dataset and, thus, cannot immediately be used. Instead, SMapper highlights the problematic designations and requests the user to either discard these entries for map generation (by not ticking them) or to select an appropriate designation from the list of available polygon names. The corrected dataset can be downloaded by the user.

## 4 Conclusion

By visually differentiating regions with missing data from those with small sample sized data, alongside frequency-interpolated areas irrespective of political borders, SMapper reduces the severe potential for misinterpretations of spatial maps produced with existing tools, which is relevant in epidemiology, anthropology and forensic applications of spatial prevalence data, including those with data limitations.

## Supplementary Material

vbad176_Supplementary_Data

## Data Availability

The data underlying this article have been compiled from published sources; see Supplementary Information for details.
