# Supplementary material for: SMapper: visualizing spatial prevalence data of all types, including sparse and incomplete datasets
Source: Bioinform Adv. 2023 Dec 1;3(1):vbad176. doi: 10.1093/bioadv/vbad176 (PMC10710370; doi:10.1093/bioadv/vbad176)

Supplementary Information

Khellaf et al.

“SMapper: Visualising spatial prevalence data of all types, including sparse and incomplete datasets”

Supplementary Methods

Input format

SMapper uses a custom and easy-to-adapt tabular file format (CSV; see Table S1 for an example) to allow automatic processing and simple updating of the used database for map generation by simple addition of new row and column entries with little processing of the data. This format allows for multiple sampling sites (columns) and features (rows). Data are specified by absolute counts. Thus, prevalence (or frequency) value specification requires counts of observation for the particular feature as well as the total sample size per site. Sites are exclusively assigned by name to polygons present in the used map data (see below); no assignment is done based on geographic coordinates. The first nine rows of the input table describe are reserved for the specification of database entries. All remaining rows contain count data to be visualized. Please note that SMapper expects the data in CSV format, with entries being separated by a comma (“,"). When using some spreadsheet software to prepare a dataset, please make sure that the comma sign is used as the separator sign when exporting to CSV. Using other separator signs will cause problems when running SMapper.

Database specification. The first nine rows of the input table assign the count data (see below) of the respective column to a continent, sub-continent, country, sub-country and sub-population (rows 1-5), followed by a polygon name (row 6). At least one entry has to be present in ‘country’, ‘sub-country’ or ‘polygon’. Entries have to match the polygon names in the map database (see below), otherwise no map will be generated. For assigning the count data to a particular polygon present in the map data (see below), SMapper follows a hierarchical bottom-up approach when more than one of these fields contain information, giving preference to more detailed information. For example, if both ‘country’ and ‘sub-country’ contain information, SMapper chooses the ‘sub-country’ entry for the polygon assignment. For historic data where the count data may refer to an area not following today’s

Supplementary Table S1: Generic input data file format example.

**Continent**	Continent 1	Continent 1	Continent 2	Continent 2
**Sub-Continent**	Sub-Continent 1	Sub-Continent 1	Sub-Continent 2	Sub-Continent 3
**Country**	Country 1	Country 1	Country 2	Country 3
**Sub-country**				Sub-Country 3
**Sub-population**	Sub-Population 1	Sub-Population 2		
**Polygon**			Polygon-name 1	Polygon-name 2
**Sample size**	80	100	50	10
**Reference**	*Reference 1*	*Reference 1*	*Reference 1*	*Reference 2*
**Comment**				
Y-haplogroup E-M75	70	60	10	10
Blue eye color	10	25	0	
Feature 3	0	15	25	
Trait 4			15	0

Please note that entries and counts have been entered for illustrative purposes only and do not reflect actual published data. Rows 3, 4 and 6 (marked in dark blue) are used for polygon assignment, with precedence given to entries in "Polygon" to those in "Sub-country" to those in "Country". *Zero entries* (marked in light green) denote investigated but not observed features at a particular sampling site (zero-frequency estimate; ZFE). *Empty fields* (marked in dark green) denote features not investigated at the particular sampling site, i.e., missing data (NA). Prevalence or frequency values are obtained as the ratio between entries in the feature/trait row and the total sample size (row 7). SMapper expects the dataset in CSV format, with comma (",") as the separator sign.

political borders, we included the polygon field. Here, a comma-separated list of geographic areas contained in the map database can be inserted to allow for manual reconstruction of the desired area. Row 7 contains the total sample size for the respective sampling site. This number is essential to calculate prevalence or frequency values from the count data in the rows below; non-specification will lead to a map generation error. Rows 8-9 can contain the site's publication reference(s) and further comments but are ignored by SMapper.

Count data. From the 10th row on, each row specifies a feature or trait that is to be illustrated. For each feature (corresponding to a particular row), a separate map is automatically produced. For example, if four trait rows are present, four separate figures will automatically be produced, with files names corresponding to the entry in the first column. Prevalence or frequency values are calculated as the ratio of the count and the total sample size specified in row 7 of the same column. To allow for differentiation between missing data and zero-frequency estimates (ZFE), the fields of features not investigated at a particular sampling site (i.e., a particular column) have to be left *blank* to indicate missing data (NA) while fields of

features investigated but not observed have to contain a *zero* to indicate a ZFE. For example, if a marker has been genotyped at site A but not at site B and the allele of interest has not been observed at site A, the field for site A has to contain a zero whereas the field for site B has to be left blank. Further sampling sites are simply added by additional columns. If an assigned polygon name (see above) appears more than once in the table, the corresponding values are automatically combined, alleviating the need for further manual processing.

Map data.

For all map generations, we used the public domain polygon vector map data provided by Natural Earth (https://www.naturalearthdata.com), namely the large scale (1:10m) cultural vector shapefiles “Admin 0 – Countries”, “Admin 1 – States, Provinces” and the map units and subunits shapefiles of the pack “Admin 0 – Details” (version 4.1.1; last accessed June 2020). These data are in ESRI shapefile format and are annotated with a large variety of attributes. Most of the data is attributed to modern political areas such as countries, states or cities.

Algorithmic details on interpolation and smoothing

SMapper overlaps two different visual scales to separate non-zero frequency (NZF) feature estimation from regions where a feature had been investigated but not observed.

Missing information. Areas that are not covered by the sampling sites provided by the input file are marked by bold hachures that strongly signal absence of data instead of an easily ignorable homogeneous background. Such pattern indicates that those regions are indeed not covered by the available data sources and no prediction or estimation of prevalence values is possible for these areas with any confidence. This feature is in striking contrast to commonly used tools that usually do not visually differentiate between missing information and zero frequency estimates (ZFE) and may, thus, be misleading in their interpretation. This holds true in particular for sparse data sets that do not provide good worldwide or even local coverage.

Interpolation and smoothing of NZF values. Very localized visualization of data points, perhaps oriented towards cities or even the centre point within some country or sub-country unit, can lead the wrong impression of a very localized occurrence of the respective feature, especially with sparse data and small sample sets for low-frequency features. In order to avoid such misinterpretation, SMapper intentionally smooths data values around and between sampling

sites, thereby “blurring” the exact location of the supporting data points for the map interpolation. To this end, we used area polygons as a basis, increasing their size and slightly smoothing the edges, during the generation process. This slightly expanded area was used to create a stack of multiple polygons with decreasing size but increasing associated frequency value from the center to the edges, therefore creating a semi-continuous fading-out effect. The number of layers is determined by the surface area of the original polygon and the corresponding frequency value (estimate). A reduced opacity was used to increase the color intensity of overlapping high frequency areas compared to high and low frequency mixtures. This approach also ensures that hachures indicating missing data will become more visible towards the edge of the interpolated area if overlapping with areas without data.

The area size of a polygon and its frequency value contribute to the number of layers via a function $L = \left(\sqrt[3]{A}\,\frac{\beta + \lceil (r * \beta) \rceil}{2\beta}\right)$ where *A* denotes the surface area of the polygon, *r* the frequency value associated with the region and β an arbitrary integer. The cube root is applied to the area values in order to reduce their variance. This area value is further scaled by the frequency value, with a frequency of 1.0 meaning no further scaling at all. The minimum number of layers a polygon comprises was set to 3 by adding a constant value of two to the result of *L*. Function *L* further determines how the sizes of the polygons are scaled: The largest layer is scaled in its x and y dimensions via a scaling factor of $s * \left(\frac{2+\mathrm{ceil}\left(\sqrt[3]{A}\right)}{L}\right)$ where *s* is a base scaling factor that was set to 1.2 in the current build. This formula causes smaller regions to be upscaled, while regions with a larger surface area will be scaled with smaller values. This ensures that the enlargement effect is visible for smaller polygons while not overly increasing the size of larger ones. Consecutive smaller polygon layers are generated by subtracting 1 from the nominator until it reaches the value 1. Edge smoothing is performed by applying the geopandas.GeoSeries.buffer function twice, once with a distance value of 0.8 and a second time with a distance value of -0.8. All value settings presented here were subjectively chosen to optimize the visual experience of the generated output but can be altered in the code if so desired.

Interpolated NZF estimates are coloured along a bright yellow (nearly zero frequency) – dark red (maximum frequency) gradient. The maximum frequency can be adapted to the data set; it is set to unity by default.

Interpolation and smoothing of ZFE values. Zero frequency estimates (ZFE) frequently occur with small sample sizes, in particular if the true frequency of the feature is low. To visualize the uncertainty pertaining these estimates and to avoid the spurious impression or misinterpretation that the feature is certainly absent from that particular area, we visualized a reasonable upper limited for the frequency instead of the zero-frequency itself. Broadly following previous suggestions for database searches in forensics and beyond (Andersen and Balding, 2021; Brenner, 2010; Robbins, 1968), we depict the inverse of the number of samples as an upper limit for the unknown feature frequency. Interpolation is done along the same lines as for the NZF case. Interpolated ZFE estimates are coloured along a black (nearly zero upper limit) – white (upper limit much larger than zero) gradient. Absent features in large sample sets are, thus, coloured in very dark colours that are similar to the background colour also used for missing data but without hachures, thereby visually indicating high confidence in the absence of that particular feature. ZFE from small sample sets will result in bright white colours, indicating that an occurrence of the feature cannot be ruled out with certainty in those areas.

Implementation.

SMapper was written in Python 3.11.10 and is therefore independent of the operating system. Aside from standard libraries always included in Anaconda3 v22.11.0, SMapper requires the libraries Geopandas v0.12.1 for the processing of the shapefiles, ttkwidgeds v0.10.0 for certain GUI functions and cartopy v0.18.0 for certain geospatial processing tasks. Different versions of these libraries might also work; however, they were not tested.

Polygon assignment.

SMapper allows easy manual and automated correction of polygon names not correctly specified in the input file. This is achieved by providing the user with an easy-to-use GUI in which the tabular data file can be edited. Upon opening, the GUI compares the three fields important for the generation of the maps (Country, Sub-Country, Polygon) to the region names in the Natural Earth database. Entries for which the most specific given descriptor field was not found in the database are marked in red. All rows can be edited via a text field that displays all available options from the database containing the currently entered string. For

the Polygon field, sub-regions can be added, removed and edited individually. Before starting the map generation process, a subset of database entries can be selected for the generation, for instance only rows not marked in red.

Multi-threading

The built-in multiprocessing module of python 3 is used to allow for the speed up of calculations. The software automatically utilizes all available CPU cores to enable a fast map generation. This is especially useful with computers that allow for numerous processes to be run in parallel.

Output format

SMapper produces graphic files in png and in pdf format, separately for each feature listed in the input file from row 10 onwards. Each map is complemented by two layer-specific legends for zero frequency estimates (ZFE; black-to-white gradient) and non-zero frequencies (NZF; yellow-to-red gradient), respectively, and a designation of areas with missing data. All produced graphic files are combined in a single zip file for convenience.

Web implementation

Web-SMapper is a web service of SMapper. It uses Pandas v1.5.2, Request, Cgitb, Urllib, and Json for communication with SMapper. A key feature of Web-SMapper is its utilization of local storage, a feature provided by HTML5, to enable the user to store data locally on their client machine using JavaScript. Local storage operates on the concept of key-value pairs, with data stored as JSON strings without any expiration date. This approach eliminates the need for continuous reloading of user data from the server during web browsing, resulting in improved website performance. The Web-SMapper interface seamlessly integrates various further libraries, such as XLSX.utils, select2, and jQuery, which are optimized to handle changes and user selections efficiently. By combining local storage with Ajax calls, the application minimizes server requests, ensuring that only necessary server resources are utilized during the creation of document sets or maps. Web-SMapper organizes the steps for data entry and required results in such a structured manner that minimizes the usage of random-access

memory and server CPU computing power, leading to a smoother and more efficient user experience.

Graphical user interface (GUI).

The GUI for the stand-alone python software was created using the native python library Tk and ttkwidgets, a collection of additional widgets by various authors (https://ttkwidgets.readthedocs.io/).

Data sets for software application examples

Prevalence of Y-haplogroup E-M75 (E2). We have compiled the spatial prevalence data of human haplogroup Y-M75 (E2) (van Oven, et al., 2014) from published reports. This includes the following sources: (Abu-Amero, et al., 2009; Altena, et al., 2020; Arredi, et al., 2004; Barbieri, et al., 2012; Battaglia, et al., 2009; Bekada, et al., 2015; Brunelli, et al., 2017; Cadenas, et al., 2008; D'Atanasio, et al., 2019; de Filippo, et al., 2011; Delfin, et al., 2011; Fortes-Lima, et al., 2015; Gomes, et al., 2010; Gonçalves, et al., 2003; Gonzalez, et al., 2013; Hammer, et al., 2006; Hassan, et al., 2008; Hudjashov, et al., 2007; Iacovacci, et al., 2017; Jankova, et al., 2019; Kampuansai, et al., 2020; Karafet, et al., 2010; Karlsson, et al., 2006; Kim, et al., 2011; Lang, et al., 2019; Lappalainen, et al., 2006; Larmuseau, et al., 2015; Luis, et al., 2004; Mona, et al., 2009; Montano, et al., 2011; Naidoo, et al., 2010; Resque, et al., 2016; Rosa, et al., 2007; Sanchez, et al., 2005; Schaan, et al., 2020; Sengupta, et al., 2006; Siva, 2008; Trejaut, et al., 2014; van Oven, et al., 2014; Volgyi, et al., 2009; Xie, et al., 2019; Zalloua, et al., 2008; Zhang, et al., 2019)

Frequency of rs4988235-A allele. Spatial frequencies for the A allele of single-nucleotide polymorphism rs4988235:G>A on chromosome 2 upstream of the *LCT* gene (also reported as rs4988235:C>T), being the dominant cause for lactase persistence (Enattah, et al., 2002) during adulthood in populations of North European ancestry, were taken from Table S2 from a recent review (Anguita-Ruiz, et al., 2020). This review combined data from previous reviews (Itan, et al., 2010; Storhaug, et al., 2017) with numerous more recent data sets up to 2020 (see Table S1 in their publication). Data are provided as Supplementary Table S2.

Blue eye colour prevalence. Published prevalence data appear to be sparse. We used the data compilation from a recent review (Katsara and Nothnagel, 2019) that aimed for worldwide coverage but experienced severe limitations in published data and also the quality of some of those publications. This review eventually provided data on European and Central Asian populations. Data are provided as Supplementary Table S3.

Consanguinity estimates. Consanguinity rates were obtained from www.consang.net which hosts a large compilation of published country- and population-specific estimates. These data had previously been used for a visualization of country-specific values, also in some cases where different estimates for different geographic regions within the same country were available (Bittles and Black, 2010). We only used estimates that refer to a country or geographic region. In some instances, we dropped studies with very low samples sizes when the same region was covered by studies of larger sample size. We could not locate the data on the Russian Federation visualized in (Bittles and Black, 2010) in this data set but additional estimates for geographic regions in Southern and Eastern Africa. Data are provided as Supplementary Table S4.

Please note that some example datasets contain, for illustrative purposes, some geographic designations that are not contained in the polygon vector data set and, thus, require prior correction of the assigned polygon names using the SMapper interface.

References

Abu-Amero, K.K., *et al.* Saudi Arabian Y-Chromosome diversity and its relationship with nearby regions. *BMC Genet* 2009;10:59.

Altena, E., *et al.* The Dutch Y-chromosomal landscape. *Eur J Hum Genet* 2020;28(3):287-299.

Andersen, M.M. and Balding, D.J. Assessing the Forensic Value of DNA Evidence from Y Chromosomes and Mitogenomes. *Genes (Basel)* 2021;12(8).

Anguita-Ruiz, A., Aguilera, C.M. and Gil, A. Genetics of Lactose Intolerance: An Updated Review and Online Interactive World Maps of Phenotype and Genotype Frequencies. *Nutrients* 2020;12(9).

Arredi, B., *et al.* A predominantly neolithic origin for Y-chromosomal DNA variation in North Africa. *Am J Hum Genet* 2004;75(2):338-345.

Barbieri, C., *et al.* Contrasting maternal and paternal histories in the linguistic context of Burkina Faso. *Mol Biol Evol* 2012;29(4):1213-1223.

Battaglia, V., *et al.* Y-chromosomal evidence of the cultural diffusion of agriculture in Southeast Europe. *Eur J Hum Genet* 2009;17(6):820-830.

Bekada, A., *et al.* Genetic Heterogeneity in Algerian Human Populations. *PLoS One* 2015;10(9):e0138453.

Bittles, A.H. and Black, M.L. Evolution in health and medicine Sackler colloquium: Consanguinity, human evolution, and complex diseases. *Proc Natl Acad Sci U S A* 2010;107 Suppl 1:1779-1786.

Brenner, C.H. Fundamental problem of forensic mathematics--the evidential value of a rare haplotype. *Forensic Sci Int Genet* 2010;4(5):281-291.

Brunelli, A., *et al.* Y chromosomal evidence on the origin of northern Thai people. *PLoS One* 2017;12(7):e0181935.

Cadenas, A.M., *et al.* Y-chromosome diversity characterizes the Gulf of Oman. *Eur J Hum Genet* 2008;16(3):374-386.

D'Atanasio, E., *et al.* Rapidly mutating Y-STRs in rapidly expanding populations: Discrimination power of the Yfiler Plus multiplex in northern Africa. *Forensic Sci Int Genet* 2019;38:185-194.

de Filippo, C., *et al.* Y-chromosomal variation in sub-Saharan Africa: insights into the history of Niger-Congo groups. *Mol Biol Evol* 2011;28(3):1255-1269.

Delfin, F., *et al.* The Y-chromosome landscape of the Philippines: extensive heterogeneity and varying genetic affinities of Negrito and non-Negrito groups. *Eur J Hum Genet* 2011;19(2):224-230.

Enattah, N.S., *et al.* Identification of a variant associated with adult-type hypolactasia. *Nat Genet* 2002;30(2):233-237.

Fortes-Lima, C., *et al.* Genetic population study of Y-chromosome markers in Benin and Ivory Coast ethnic groups. *Forensic Sci Int Genet* 2015;19:232-237.

Gomes, V., *et al.* Digging deeper into East African human Y chromosome lineages. *Hum Genet* 2010;127(5):603-613.

Gonçalves, R., *et al.* Y-chromosome lineages in Cabo Verde Islands witness the diverse geographic origin of its first male settlers. *Human genetics* 2003;113(6):467-472.

Gonzalez, M., *et al.* The genetic landscape of Equatorial Guinea and the origin and migration routes of the Y chromosome haplogroup R-V88. *Eur J Hum Genet* 2013;21(3):324-331.

Hammer, M.F., *et al.* Dual origins of the Japanese: common ground for hunter-gatherer and farmer Y chromosomes. *J Hum Genet* 2006;51(1):47-58.

Hassan, H.Y., *et al.* Y-chromosome variation among Sudanese: restricted gene flow, concordance with language, geography, and history. *Am J Phys Anthropol* 2008;137(3):316-323.

Hudjashov, G., *et al.* Revealing the prehistoric settlement of Australia by Y chromosome and mtDNA analysis. *Proc Natl Acad Sci U S A* 2007;104(21):8726-8730.

Iacovacci, G., *et al.* Forensic data and microvariant sequence characterization of 27 Y-STR loci analyzed in four Eastern African countries. *Forensic Sci Int Genet* 2017;27:123-131.

Itan, Y., *et al.* A worldwide correlation of lactase persistence phenotype and genotypes. *BMC Evol Biol* 2010;10:36.

Jankova, R., *et al.* Y-chromosome diversity of the three major ethno-linguistic groups in the Republic of North Macedonia. *Forensic Sci Int Genet* 2019;42:165-170.

Kampuansai, J., *et al.* Paternal genetic history of the Yong population in northern Thailand revealed by Y-chromosomal haplotypes and haplogroups. *Mol Genet Genomics* 2020;295(3):579-589.

Karafet, T.M., *et al.* Major east-west division underlies Y chromosome stratification across Indonesia. *Mol Biol Evol* 2010;27(8):1833-1844.

Karlsson, A.O., *et al.* Y-chromosome diversity in Sweden - a long-time perspective. *Eur J Hum Genet* 2006;14(8):963-970.

Katsara, M.A. and Nothnagel, M. True colors: A literature review on the spatial distribution of eye and hair pigmentation. *Forensic Sci Int Genet* 2019;39:109-118.

Kim, S.H.*, et al.* High frequencies of Y-chromosome haplogroup O2b-SRY465 lineages in Korea: a genetic perspective on the peopling of Korea. *Investig Genet* 2011;2(1):10.

Lang, M.*, et al.* Forensic characteristics and genetic analysis of both 27 Y-STRs and 143 Y-SNPs in Eastern Han Chinese population. *Forensic Sci Int Genet* 2019;42:e13-e20.

Lappalainen, T.*, et al.* Regional differences among the Finns: a Y-chromosomal perspective. *Gene* 2006;376(2):207-215.

Larmuseau, M.H.*, et al.* The Paternal Landscape along the Bight of Benin - Testing Regional Representativeness of West-African Population Samples Using Y-Chromosomal Markers. *PLoS One* 2015;10(11):e0141510.

Luis, J.R.*, et al.* The Levant versus the Horn of Africa: evidence for bidirectional corridors of human migrations. *Am J Hum Genet* 2004;74(3):532-544.

Mona, S.*, et al.* Genetic admixture history of Eastern Indonesia as revealed by Y-chromosome and mitochondrial DNA analysis. *Mol Biol Evol* 2009;26(8):1865-1877.

Montano, V.*, et al.* The Bantu expansion revisited: a new analysis of Y chromosome variation in Central Western Africa. *Molecular ecology* 2011;20(13):2693-2708.

Naidoo, T.*, et al.* Development of a single base extension method to resolve Y chromosome haplogroups in sub-Saharan African populations. *Investig Genet* 2010;1(1):6.

Resque, R.*, et al.* Male Lineages in Brazil: Intercontinental Admixture and Stratification of the European Background. *PLoS One* 2016;11(4):e0152573.

Robbins, H.E. Estimating the Total Probability of the Unobserved Outcomes of an Experiment. *Ann Math Stat* 1968;39(1):256-257.

Rosa, A.*, et al.* Y-chromosomal diversity in the population of Guinea-Bissau: a multiethnic perspective. *BMC Evol Biol* 2007;7:124.

Sanchez, J.J.*, et al.* High frequencies of Y chromosome lineages characterized by E3b1, DYS19-11, DYS392-12 in Somali males. *Eur J Hum Genet* 2005;13(7):856-866.

Schaan, A.P.*, et al.* New insights on intercontinental origins of paternal lineages in Northeast Brazil. *BMC Evol Biol* 2020;20(1):15.

Sengupta, S.*, et al.* Polarity and temporality of high-resolution y-chromosome distributions in India identify both indigenous and exogenous expansions and reveal minor genetic influence of Central Asian pastoralists. *Am J Hum Genet* 2006;78(2):202-221.

Siva, N. 1000 Genomes project. *Nature biotechnology* 2008;26(3):256-257.

Storhaug, C.L., Fosse, S.K. and Fadnes, L.T. Country, regional, and global estimates for lactose malabsorption in adults: a systematic review and meta-analysis. *Lancet Gastroenterol Hepatol* 2017;2(10):738-746.

Trejaut, J.A.*, et al.* Taiwan Y-chromosomal DNA variation and its relationship with Island Southeast Asia. *BMC Genet* 2014;15:77.

van Oven, M.*, et al.* Human genetics of the Kula Ring: Y-chromosome and mitochondrial DNA variation in the Massim of Papua New Guinea. *Eur J Hum Genet* 2014;22(12):1393-1403.

van Oven, M.*, et al.* Seeing the wood for the trees: a minimal reference phylogeny for the human Y chromosome. *Hum Mutat* 2014;35(2):187-191.

Volgyi, A.*, et al.* Hungarian population data for 11 Y-STR and 49 Y-SNP markers. *Forensic Sci Int Genet* 2009;3(2):e27-28.

Xie, M.*, et al.* Genetic substructure and forensic characteristics of Chinese Hui populations using 157 Y-SNPs and 27 Y-STRs. *Forensic Sci Int Genet* 2019;41:11-18.

Zalloua, P.A.*, et al.* Y-chromosomal diversity in Lebanon is structured by recent historical events. *Am J Hum Genet* 2008;82(4):873-882.

Zhang, Y.*, et al.* Genetic polymorphism of 190 Y-SNPs in Han population from Jiangsu province, China. *Forensic Science International: Genetics Supplement Series* 2019;7(1):552-554.

Supplementary Figure S1. Comparative global spatial prevalence visualisations for Y-hg E-M75 (E2). A: HeatMapper Geocoordinate; **B:** HeatMapper Geomap; **C:** SMapper.

A

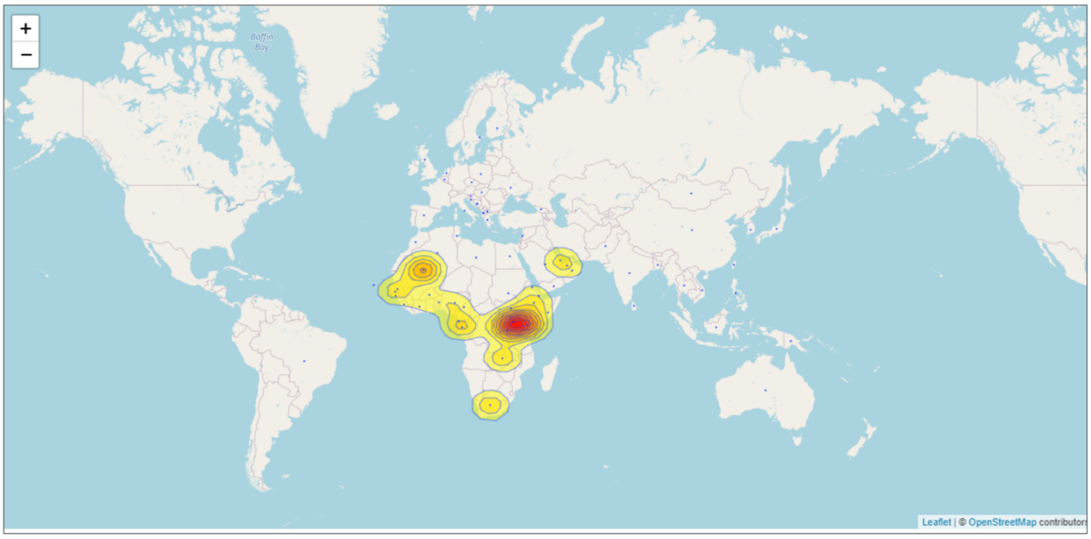

B

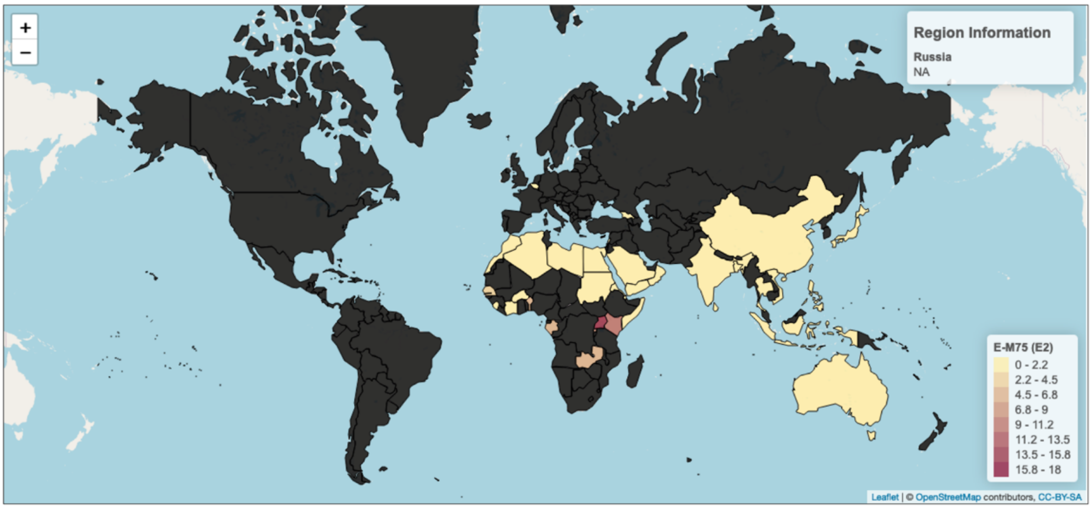

C

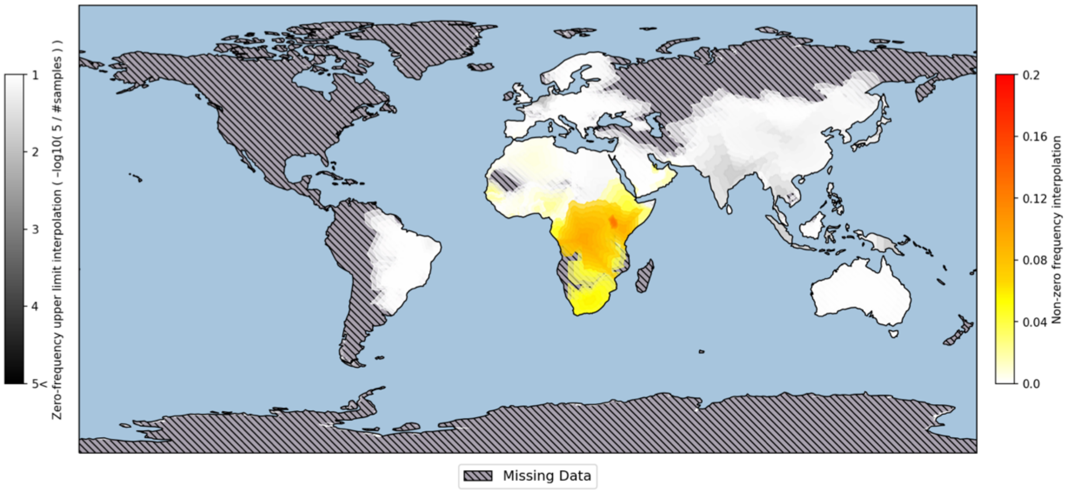

Supplementary Figure S2. Comparative global spatial prevalence visualisations for the lactase-persistence conferring SNP rs4988235 allele. A: HeatMapper Geocoordinate; **B:** HeatMapper Geomap; **C:** SMapper.

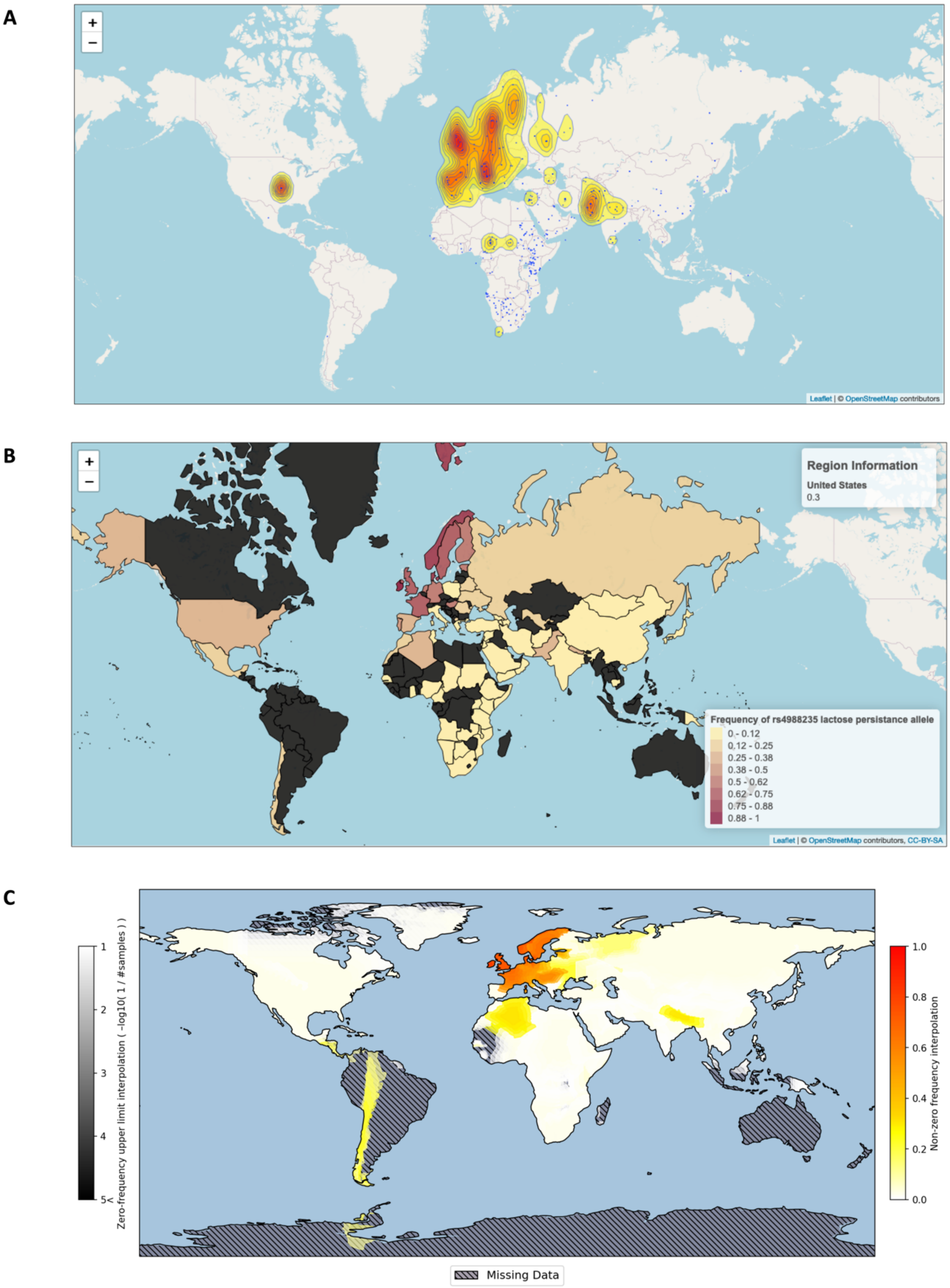

Supplementary Figure S3. Comparative global spatial prevalence visualisations for blue eye colour. A: HeatMapper Geocoordinate; **B:** HeatMapper Geomap; **C:** SMapper.

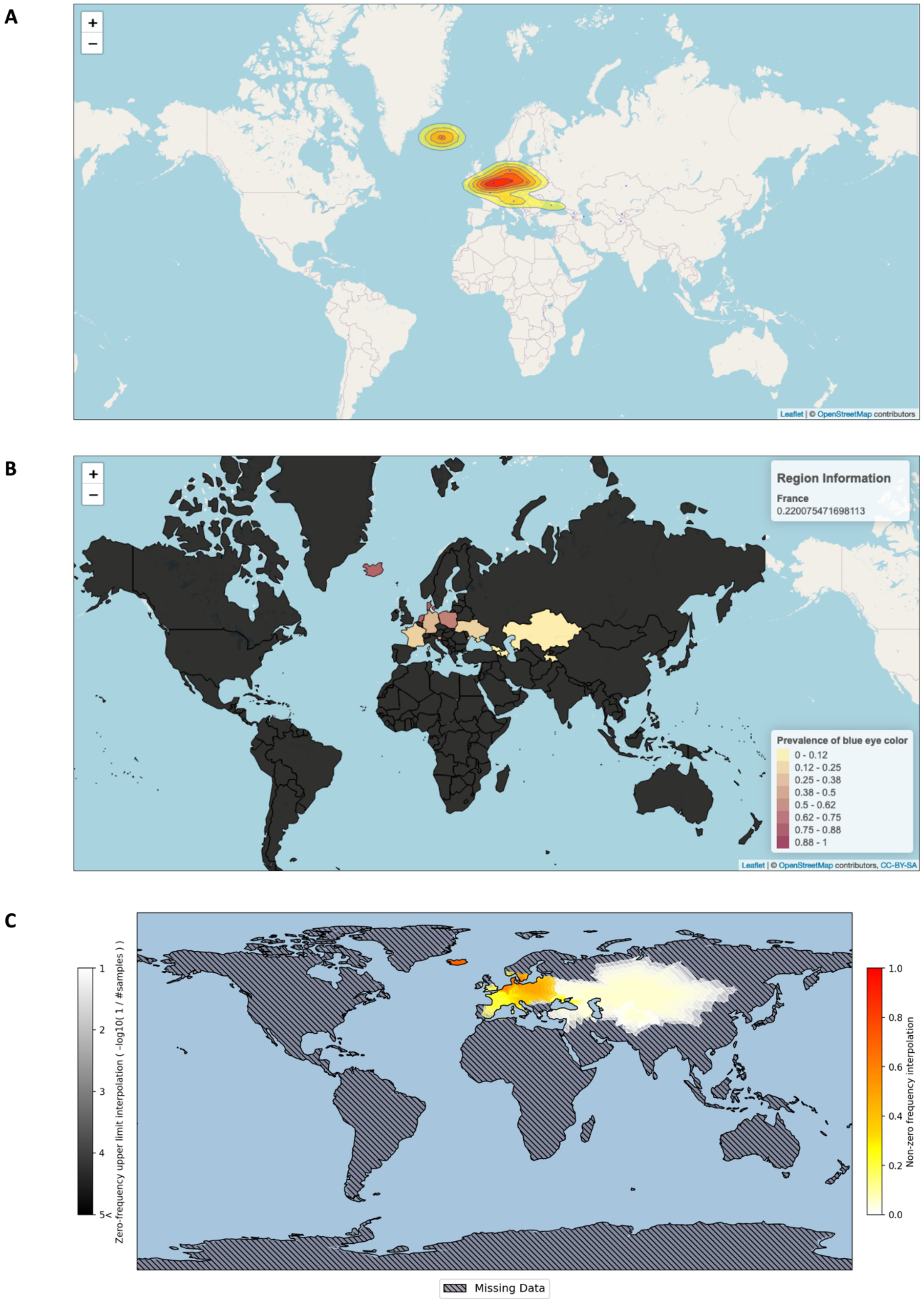

Supplementary Figure S4. Comparative global spatial prevalence visualisations for consanguinity. A: HeatMapper Geocoordinate; **B:** HeatMapper Geomap; **C:** SMapper.

A

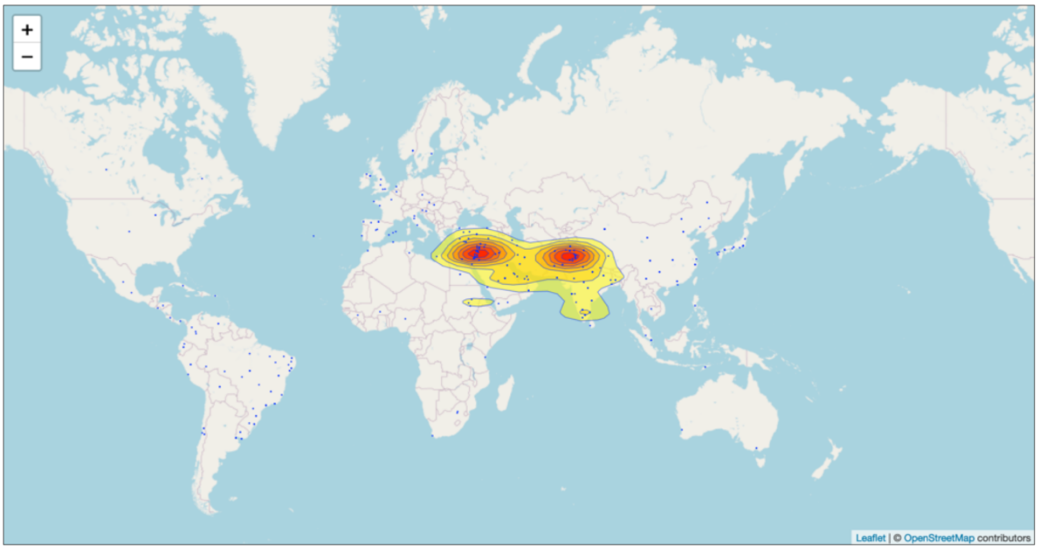

B

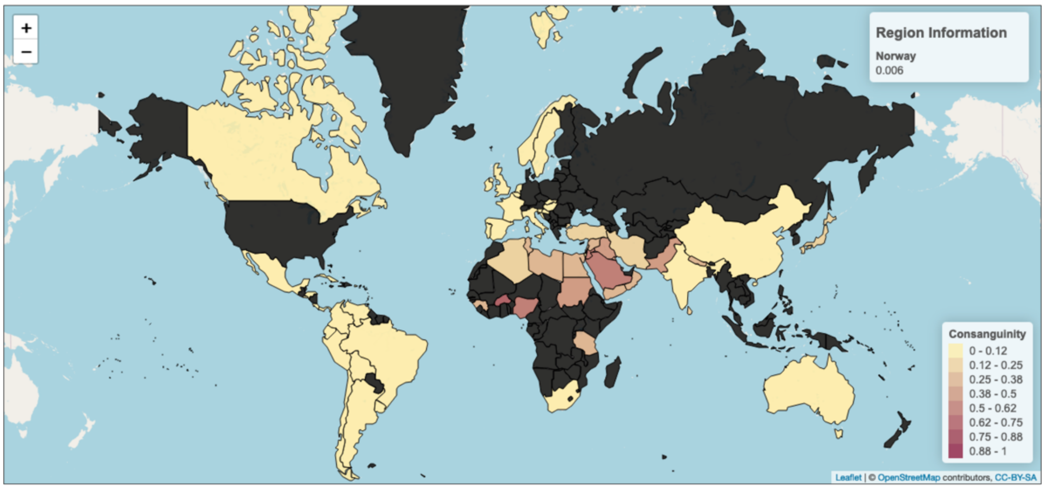

C

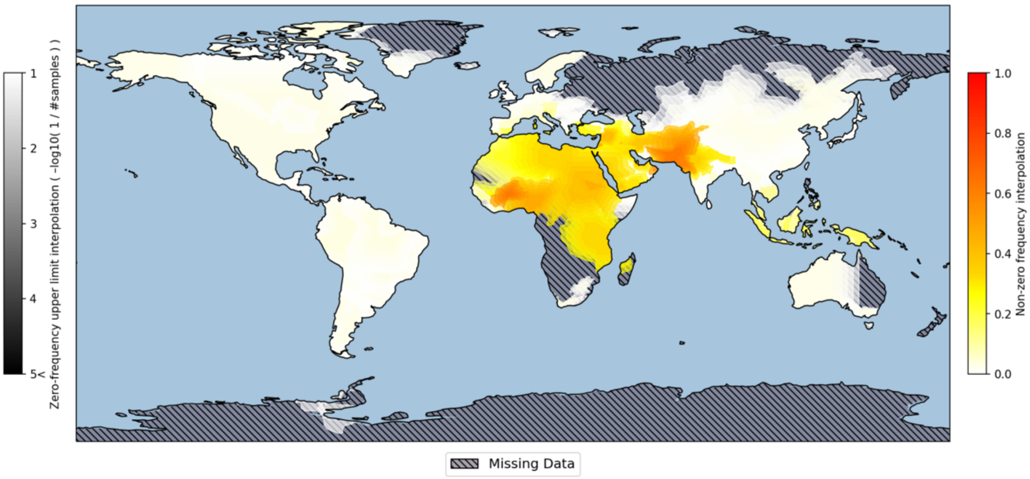

Supplement: vbad176_Supplementary_Data [file vbad176_supplementary_data.zip › SMapper_SM.pdf]
